# Experimental Study of Wheel-to-Rail Interaction Using Acceleration Sensors for Continuous Rail Transport Comfort Evaluation

**DOI:** 10.3390/s23198064

**Published:** 2023-09-25

**Authors:** Ioana Mihăilescu, Gabriel Popa, Emil Tudor, Ionuț Vasile, Marius Alin Gheți

**Affiliations:** 1 Doctoral School of FIMM, National University of Science and Technology POLITEHNICA of Bucharest, 060042 Bucharest, Romania; omidu2000@yahoo.com; 2 Railway Vehicles Department, National University of Science and Technology POLITEHNICA of Bucharest, 060042 Bucharest, Romania; gabriel.popa@upb.ro (G.P.); marius_alin.gheti@upb.ro (M.A.G.); 3 Renewable Sources and Energy Efficiency Department, National Institute for Research and Development in Electrical Engineering ICPE-CA, 030138 Bucharest, Romania; ionut.vasile@icpe-ca.ro

**Keywords:** wagon vibration, wheel-to-rail, transportation comfort, acceleration sensor, vibration detection, electronic sensors

## Abstract

Rail transport comfort is ensured by predictive maintenance and continuous supervision of rail quality. Besides the specialized equipment, the authors are proposing a simple system that can be implemented on operational wagons while in service, aiming to detect irregularities in the rail and report them using the train’s online communication lines. The sensor itself is an acceleration sensor connected to an electronic microcontroller able to filter the inrush acceleration and send it to the diagnosis system of the wagon. This paper presents a study of real data recorded of the transversal and vertical vibrations of a standard tank wagon, measured on 2 axles and the car body, followed by the interpretation of the recorded data.

## 1. Introduction

The wheel-to-rail interaction is a specific issue for railway transportation means, regarding wear, fault anticipation, comfort, and safety. All of them are highly documented in the literature regarding the vibrations of the railway vehicles [1], the dynamic response in the rail vehicles to the track vertical irregularities [2,3], and more often after the speed was increased on the railway.

The wheel-to-rail interaction is the subject of [4], where an investigation of the dynamic effects of a wheel running on a discretely supported rail was investigated [5].

The suspension of the wagon is analyzed and optimized using dedicated models [6] and implementing the simulations of track irregularities [7], the modeling of the railway bogie [8]. Some applications can be found in [9], and a complex review of the modeling methods for railway suspension components [10] can be used for the optimization of the bogie frames and suspension, as testing them must be performed after the results of the calculations are validated at safe speeds.

Measuring the vibrations induced in the car body by the track unevenness [11] is performed using accelerometers mounted on the car body, analyzing the amplitude of the acceleration and the frequency of the signal. In [12], researchers present a numerical analysis regarding the correlation between the dynamic response of a bogie and the displacements of the axles during circulation on a track with vertical irregularities in a harmonic behavior of vibration, a method that can be used to diagnose the faulty dumpers of the suspension.

The higher levels of acceleration can be measured by placing an acceleration sensor on the axle box. The operation is intended to measure the efforts on the axle and bearings. The paper [13] analyzes the correlation between axle box accelerations in lateral and vertical directions and differently processed track geometry parameters based on an accurate measurement run on a straight track.

The electronic sensors used for acceleration measuring can be of the piezoelectric type [14,15], one-direction sensors [16], or the newest three-axis accelerometers with micro-mechanical (MEM) technology, a sensor that eliminates the need for selection, qualification, and system-level integration of discrete devices. The study [17] investigates the development of a nanocomposite that can exhibit both piezoelectric and magnetic effects in sensing and actuation, respectively.

The application of acceleration sensors in the measurement of track irregularities is presented in [18], focused on an irregularity probabilistic model that lays the foundation for stochastic and probabilistic analysis of vehicle/track systems.

A similar approach to using acceleration sensors on axle boxes is presented in [19], where there is a comparison between irregularities provided by rail joints and welded rail. A similar method was used in [20], regarding the correlation between vehicle responses and track irregularities using dynamic simulations and measurements.

The localization of the track irregularities is presented in [21] by using the time and distance measurements.

In [22], there are measurements of track irregularities presented in graphs related to distance, with a precision of 3–25 m, for a locomotive and a passenger wagon, aiming to use the same data recorded for all the vehicles as being correlated.

The development of a numerical model for a railway vehicle’s comfort assessment through comparison with experimental measurements [23] presents a solution in which they compare the measurements alongside the car body and state that the values read near the bogies are close to the real ones, while the sensors placed in the middle of the vehicle are subject to noise by adding the influences of both bogies.

An interesting article [24] presents an experiment with some low-cost sensors placed alongside a train in which data were collected from nine sensors to calibrate the information they provided and to evaluate the comfort of the passengers using the RMS average value for the acceleration.

The present paper will present in Section 2 the experimental setup for collecting acceleration information from one tank wagon, unloaded, and loaded, performed on an ellipsoid track, part of the test facility located in Faurei, Romania.

The data collected were presented in Section 3 and analyzed to detect that the values are correct and to decide whether the acceleration measured on the car body is clear and how relevant the values are for characterizing the comfort of the passengers.

The purpose of this study is to determine the optimal number and best localization/positioning of acceleration sensors that are to be used in a future research project, in cooperation with the train’s operator, to supervise the quality of the ride, the comfort of the passengers, and, mostly, the safety issues that can appear during normal operation of the trains.

## 2. Materials and Methods

The tests were performed in the test ring of the Romanian Railway Authority (AFER), which is the railway and subway specialized technical body of the Ministry of Transport and Infrastructure. The main ring has the following characteristics [25]:

Total length of lines: 20.2 km, interlocking system, from which:

- Big ring, with the following characteristics:
- 13.7 km with 6 footbridges and 4 level crossings;
- maximum speed 200 km/h;
- two curves with a radius of 1800 m and the cants of track of 150 mm;
- length of the straight lines 1000 m, 950 m;
- electrification in single-phase alternating current of 25 kV/50 Hz.

Small ring, with the following characteristics:

- 2.2 km with 5 footbridges, non-electrified;
- maximum speed 60 km/h;
- curves with a radius of 800 m, 400 m, 250 m, 180 m and guard of track of 70 mm and 130 mm.

Twisted test line with curves and reverse curves of variable radius: with the smallest radius 135 and the biggest radius 250 on a length of 765 m.

Collision line with hump.

Hall of 600 m^2^ with inspection pits, lifting jacks 4 × 20 t and 3.2 t cranes (inside the hall) and 6.2 t (outside of the hall).

Building with offices and accommodation rooms having a total area of 583 m^2^ (6 double rooms), meeting room (for about 20 persons), dining room, and kitchen.

The tests can be performed to measure the horizontal-longitudinal and horizontal-transversal accelerations for assessing the quality of running of railway vehicles.

The standard EN 14363:2016 [26] has defined the following notations:

- lateral movement (acceleration): y;
- vertical movement (acceleration): z;
- longitudinal movement (acceleration): x.

### 2.1. Selecting the Acceleration Sensors (Material, Range)

Selecting the acceleration sensors must consider the measuring range, the maximum non-destructive shock, the mechanical dimensions, mass, connection type, and the type of mounting. Accuracy is related to the signal’s frequency, which depends on the operational speed of the vehicle.

#### 2.1.1. The Measuring Range and the Accuracy

According to the study described in the introductory chapter, the selected sensor will measure the axle acceleration, and the measured acceleration can be higher than 20 m/s^2^. The selected accelerometer must be able to measure such acceleration; thus, the version we have selected has a voltage sensitivity of 1 mV/ms^−2^ with 5% accuracy.

#### 2.1.2. The Maximum Non-Destructive Shock

For the maximum non-destructive shock, the sensor must resist accelerations up to 500 m/s^2^, and we must select a sensor with a maximum operational continuous sinusoidal acceleration (+/− peak) of 7000 m/s^2^.

#### 2.1.3. Mechanical Characteristics

For the mechanical characteristics, we must select the weight of the sensor (it must be as light as possible to have small inertia), and values range from 4.8 to 6.5 g.

Moreover, we must select the connection type—one electrical connector is axial (aligned with the sensor’s sensing axle).

Another specific detail regarding sensors is the mounting of the sensor on the body of the vehicle. In this case, some sensors can offer some mounting accessories, such as clips, which can be welded, glued, or fixed with a screw. This solution is recommended when using expensive sensors in a rushed environment.

The selected sensor is Hottinger Bruel Mechanic 1-B12/500, with the specifications presented in Table 1.

### 2.2. Mounting the Acceleration Sensors on the Bogie and the Car Body

**Figure 1:**
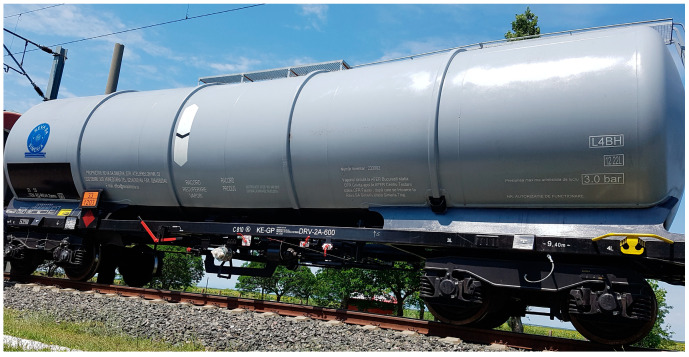
Tank wagon used during the tests.

The car body detail of the tank wagon is presented in Figure 1, and the forward towing direction is toward the left.

The sensors were screwed onto metallic mounts, which were welded to the body frame. The sensors can be easily removed without damaging them or the body frame of the wagon after tests.

#### 2.2.1. Mounting the Acceleration Sensors on the Bogie

**Figure 2:**
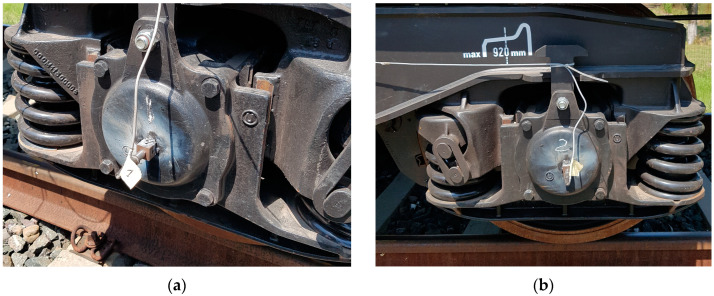
Mounting accelerometers on the axle of the first bogie: (**a**) accelerometer mounted on the axle 1; (**b**) accelerometer mounted on the axle 2.

On bogie 1 of the couch, we mounted two sensors on the axle’s cover, one for axle 1 and the other on axle 2 of the same bogie. Each sensor is dedicated to recording lateral acceleration. The sensor’s clips were welded, as in Figure 2.

#### 2.2.2. Mounting the Acceleration Sensors on the Car Body

**Figure 3:**
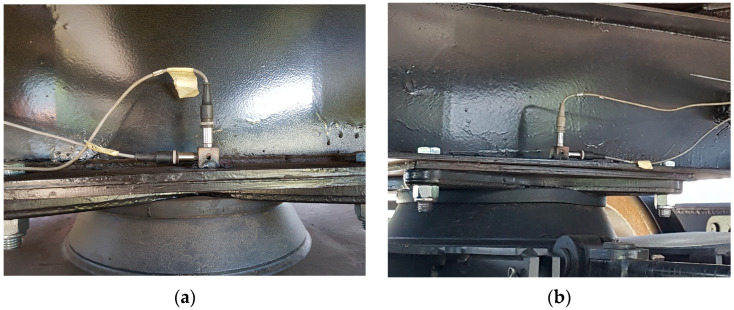
Mounted accelerometers on the frame: (**a**) near the first bogie; (**b**) near the second bogie.

We mounted four sensors on the couch, two on the frame near the bogie 1, and two on the frame on the bogie 2. Each sensor was used for recording the transversal and vertical acceleration, which are essential for derailment prevention. The sensor’s clips were welded, as in Figure 3.

As a recap, we have mounted six sensors: two measuring transversal acceleration on the axle cap on the first bogie, two measuring the frame’s transversal acceleration, and two measuring the vertical acceleration on the frame, near each bogie.

### 2.3. The Hardware for Reading the Sensor’s Output

The device used for reading the sensor’s output is a measuring amplifier system type MGC plus, produced by Hottinger, having the characteristics described in Table 2.

The values to be measured are voltages, and the acceleration can be computed using the conversion ratio from Table 1.

The six acceleration transducers are connected each to an input of the MCG amplifier and stored on external memory for future analysis. Data are saved in the numeric format and can be evaluated using spreadsheet software, (e.g., MS Excel 2007).

### 2.4. Software Developed for the Experimental Model

The recording software developed for the experimental model is a dedicated record-oriented software program built into the MCG amplifier, which stores the recorded data on a portable memory by writing a file with the extension *.tsx, which is, in fact, an *.xls with data stored on rows, while the column contains the following variables:

- Time (×0.02 s);
- Acc1 (in V)—transversal acceleration 1 from bogie 1—in m/s^2^;
- Acc2 (in V)—transversal acceleration 2 from bogie 1—in m/s^2^;
- Acc3 (in V)—horizontal acceleration from bogie 1—in m/s^2^;
- Acc4 (in V)—vertical acceleration from bogie 1—in m/s^2^;
- Acc5 (in V)—horizontal acceleration from bogie 2—in m/s^2^;
- Acc6 (in V)—vertical acceleration from bogie 2—in m/s^2^;

The first lines of the recorded file contain a description of the sensors used, the conversion ratio, and the speed of the train during recording. An example of the file content is presented in Table 3.

One can observe that the Acc1 and Acc2 columns have similar values, as the current recording was performed when the wagon is running 120–180 km/h in a curve, and the values correspond to the centrifugal acceleration, with the second axle having stronger acceleration than the first one. The same solicitation is applied to the sensors providing the Acc3 and Acc5 values, both being positive and near the 1 m/s^2^ value, but the first bogie has stronger acceleration.

## 3. Results

### 3.1. The Presentation of the Rough Data Saved during the Tests with the Unloaded Wagon

Tests were performed on the Făurei rail ring, with the unloaded wagon being trailed by a high-speed locomotive at a constant speed between 120 and 135 km/h, and records are trying to read the lateral solicitation of the components of the suspension—the first two axles located on the first bogie, and the acceleration on the frame of the wagon, first two accelerometers on the frame near the bogie 1, and the last two accelerometers on the frame, near the second bogie. The following figures present the raw data and are designated to evaluate the maximum accelerations for the characterization of the wagon’s stability.

#### 3.1.1. Acceleration Measured at the Axle

**Figure 4:**
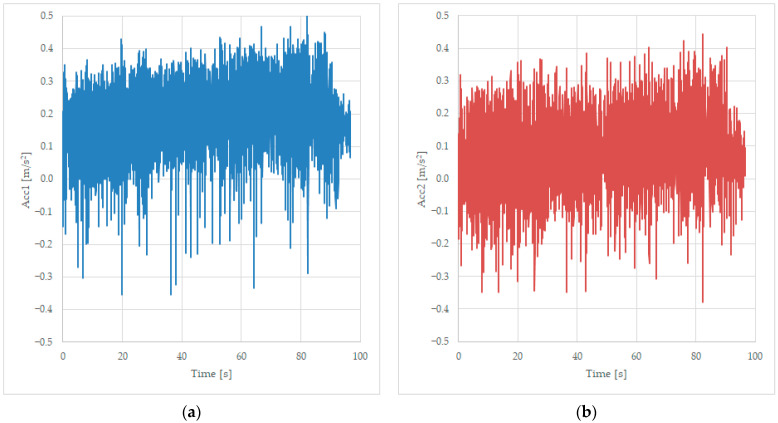
Transversal acceleration was recorded while running on a curve at 125 km/h, on the first axle of the bogie 1 (**a**) and on the second axle of the bogie 1 (**b**).

Tests were completed using the unloaded wagon at a constant speed of 125 km/h, and records are trying to read the lateral solicitation of the components of the suspension—the first two axles located on the first bogie, identified as Acc1 and Acc2, as presented in Figure 4.

**Figure 5:**
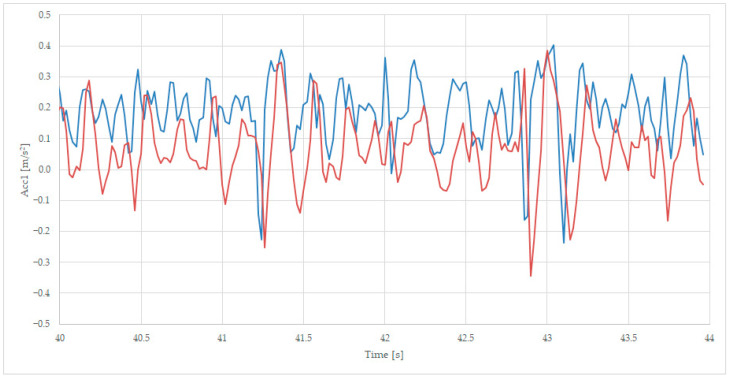
Transversal acceleration was recorded on the first axle of bogie 1 (blue) and the second axle of bogie 1 (red), while on curve, at 125 km/h.

The information is very important to measure the behavior of the rolling equipment during curve passing because major lateral acceleration can produce derailments. In Figure 5, we have selected both accelerations on the same graphic.

The acceleration from the first sensor (the blue line) is duplicated in a short time (0.02–0.04 ms) by the second sensor information (the red line), as can be seen in Figure 5, because of the irregularities of the rail, which are first visited by the wheels of axle 1 and, later, by the wheels of axle 2. Moreover, in the figure, can be observed that the irregularities are producing bilateral acceleration of +/−0.3 m/s^2^, which can be superposed on the average value of 0.1 m/s^2^, a value that corresponds to the centrifugal force during the curve passing, diminished by the gravitational component imposed by the lateral slope of the rail in the curve. At the moment, at 41.3 s and 42.8 s, the rail has some minor irregularities (joints) that are inducing a small lateral acceleration of 0.3 m/s^2^, which is the normal answer for a new suspension and an unloaded wagon.

#### 3.1.2. Acceleration Measured on the First Bogie

**Figure 6:**
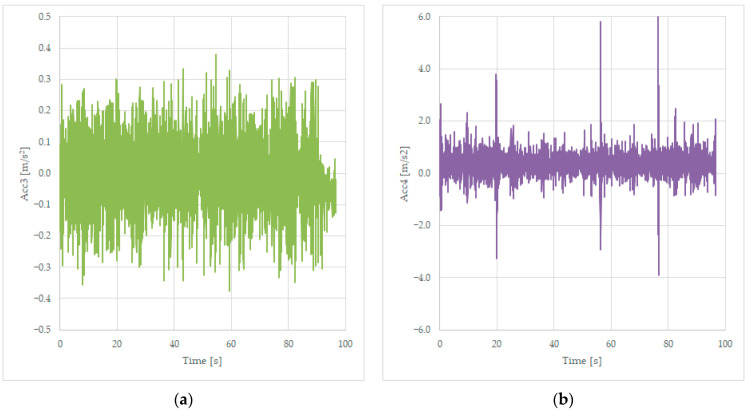
Transversal acceleration (**a**) and vertical acceleration (**b**) were recorded while running on a curve at 125 km/h, on the frame, near bogie 1.

Several tests were performed, and data were recorded in the same file as the tests from Section 3.1.1, as presented in Table 1, in the Acc3 variable for the transversal acceleration of the frame of the wagon near bogie 1 and stored in the Acc4 variable for the vertical acceleration measured on the same place on the wagon, as presented in Figure 3. Graphs are presented in Figure 6.

The lateral acceleration is close to the one recorded by the sensors mounted on the axles and presented in Figure 4. But the vertical acceleration has bigger values, up to 6 m/s^2^, which is much more important than the fluctuation of the transversal acceleration of 0.4 m/s^2^. The three peak values of the acceleration recorded in Figure 6b correspond to three joints on the line, these being the irregularities of the line.

#### 3.1.3. Acceleration Measured on the Second Bogie

**Figure 7:**
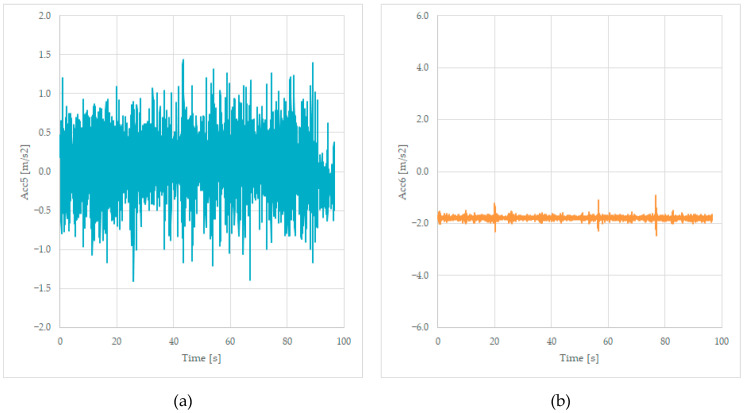
Transversal acceleration (**a**) and vertical acceleration (**b**) were recorded while running on a curve at 125 km/h, on the frame, near the bogie 2.

We will present the last two signals measured, the transversal acceleration Acc5 and the vertical acceleration Acc6, measured on the frame of the wagon, near the bogie 2. The values are stored in columns 6 and 7, as presented in Table 1, and the graphs are shown in Figure 7.

The accelerations measured near the second bogie reveal transversal values of −1 up to +1.2 m/s^2^, values that have an average value of 0.3 m/s^2^, which is like the previously measured value based on Acc1, Acc2, and Acc3 recorded data. The Acc6 values are negative in this case of an unloaded wagon because of the suspension of the wagon. We will observe in Figure 7b the presence of the three peak values of the track irregularities as in Figure 6b.

#### 3.1.4. Interpretation of the Measurements

The measurements must be compared and analyzed to decide which measuring point from the six proposed ones can be recommended for dedicated measurements, such as measurements regarding the lateral stability of the wagon, the derailment speed limit prediction, and the vertical load of each bogie.

For lateral stability analysis, we propose to superpose the transversal accelerations Acc1, Acc2, and Acc3 values in one graph, near the point of the maximum amplitude of the recorded accelerations, as presented in Figure 4 and Figure 6a.

**Figure 8:**
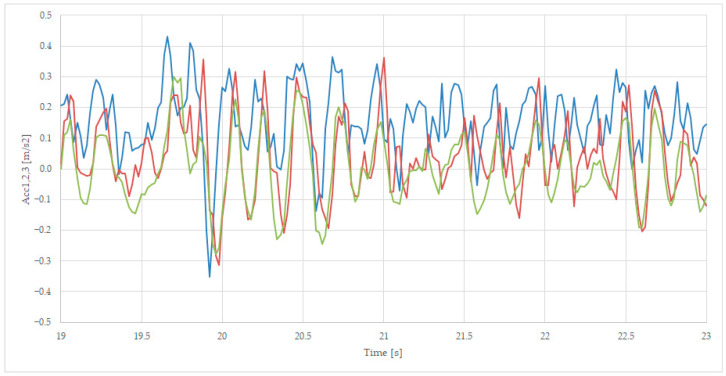
Comparing transversal acceleration on sec 19 (axle 1—blue, axle 2—red, and body—green), for unloaded wagon.

**Figure 9:**
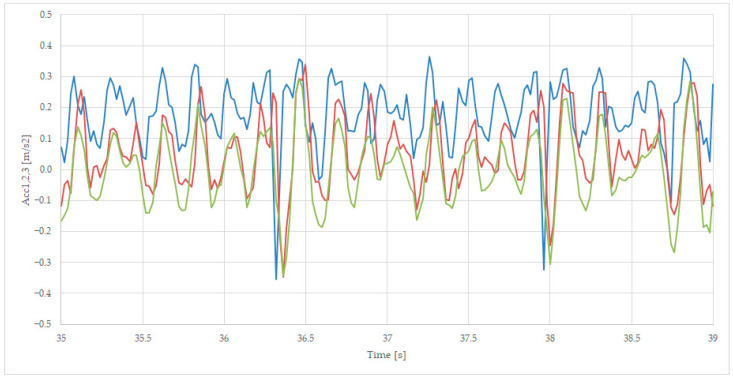
Negative peak transversal acceleration, sec 35 (axle 1—blue, axle 2—red, and body—green).

**Figure 10:**
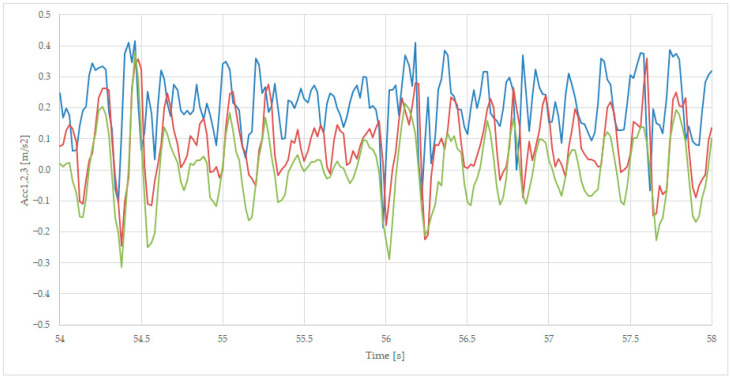
Transversal acceleration on rail joint, sec 54 (axle 1—blue, axle 2—red, and body—green).

We presented the details in separated figures, by zoom in on the points 19–23 s for Figure 8, 36–40 s for Figure 9, and 54–58 s for Figure 10, where the colors of the variables are Acc1-blue, Acc2—red, and Acc3—green.

Figure 8 presents the maximum positive point of the first axle acceleration Acc1 at moment 19.7 s, with a value of 0.42 m/s^2^, and 19.8 s with a value of 0.41 m/s^2^, the latter being followed by a maximum negative acceleration at moment 19.8 s, with a value of −0.35 m/s^2^. The Acc2 signal is following the Acc1 signal with a 0.1 s delay because of the distance between the two axles. The Acc3 signal has slightly smaller values, also being delayed by 0.05 s from Acc2, due to the suspension’s tampering action.

Figure 9 presents the maximum negative accelerations at moment 36.3 s, with a value of −0.35 m/s^2^, and at moment 36.3 s, with a value of −0.32 m/s^2^. The Acc2 signal is following the Acc1 signal with a 0.1 s delay because of the distance between the two axles. The Acc3 signal has similar negative values as the Acc2 ones, showing that the suspension operates less in this case.

Figure 10 presents the behavior of the wagon when passing rail joints, with all three accelerations presenting similar values, such as 54.4 s, with values from −0.3 m/s^2^ to 0.4 m/s^2^, and 56 s with values from −0.3 m/s^2^ to 0.4 m/s^2^. The Acc1 positive values of the acceleration are bigger than the values for Acc2, mainly because the first axle is the first to attach the line irregularities. The Acc3 signal has a bigger amplitude for negative accelerations because of the inertia of the frame.

### 3.2. The Presentation of the Rough Data Saved during the Tests with the Loaded Wagon

The same tests as described in Section 3.1 were performed with the loaded wagon being trailed by a high-speed locomotive at a constant speed between 100 and 120 km/h, and records are to be compared with the initial test with the unloaded wagon. The analyzed wagon is a tank for fluids, and the influence of liquid inertia and viscosity can be considered when analyzing the accelerations of the car frame. The following figures present the raw data and are designated to evaluate the maximum accelerations for the characterization of the wagon’s stability.

#### 3.2.1. Acceleration Measured at the Axle for the Loaded Wagon

**Figure 11:**
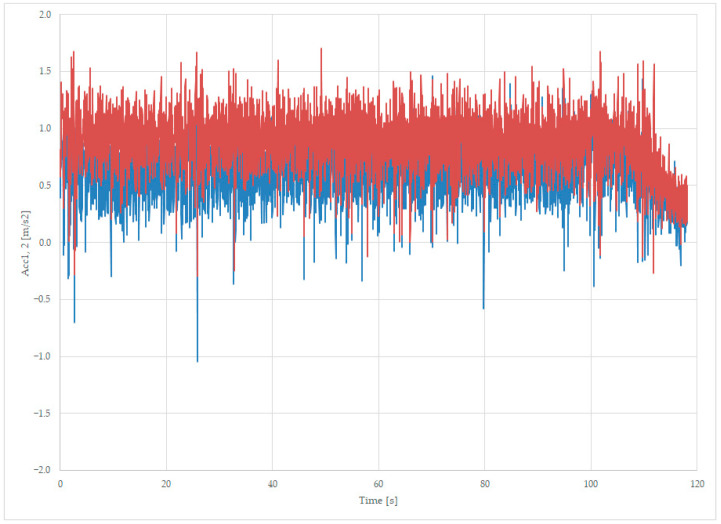
Transversal acceleration was recorded while running loaded on a curve at 110 km/h, on the first axle of bogie 1 (blue) and the second axle of bogie 1 (red).

Tests were completed using the unloaded wagon at a constant speed of 110 km/h, and records are trying to read the lateral solicitation of the components of the suspension—the first two axles located on the first bogie, identified as Acc1 and Acc2. In Figure 11, there are the first two signals measured: Acc1 from axle 1 and Acc2 for the second axle of the first bogie.

Both accelerations measured present a centrifugal component of the acceleration, with similar values, despite the lateral slope of the rails in the curve. For the unloaded wagon, in Figure 4, there is an average value for acceleration near 0 m/s^2^, while for the loaded wagon, the average value is about 0.8 m/s^2^.

The data presented in Figure 11 are recorded with a loaded wagon on a curve with a radius of 1800 m and lateral slope, crossed with a constant speed of 110 km/h, and it is normal to measure a non-zero value of acceleration because of the difference between the centrifugal acceleration and the normal component of the gravitational acceleration, thus making the trend of these measurements to be 0.635–0.884 m/s^2^. The final part of the record, from 100 s to 116 s, represents the ending of the curve with a decreasing average acceleration.

**Figure 12:**
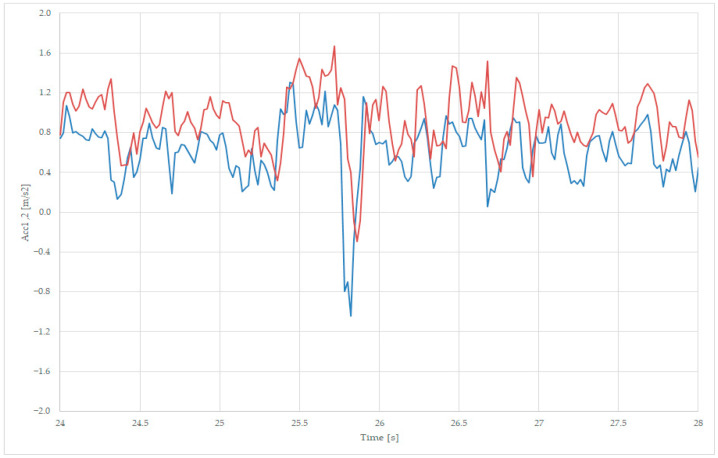
Transversal acceleration was recorded on the first axle of bogie 1 (blue) and the second axle of bogie 1 (red), while loaded, on a curve, at 110 km/h.

The loaded rolling equipment during curve passing has three times more important lateral acceleration, which can lead to derailments (see Figure 12), in which we have selected both accelerations measured on the axles of the first bogie on the same graphic, zoomed near a negative spike at the moment 25.8 s.

The positive values are close and have the same trend, with a small gap dictated by the bogie length, while the negative spikes of 1 m/s^2^ are more accentuated on the first axle but still under the positive values.

#### 3.2.2. Acceleration Measured on the First Bogie

**Figure 13:**
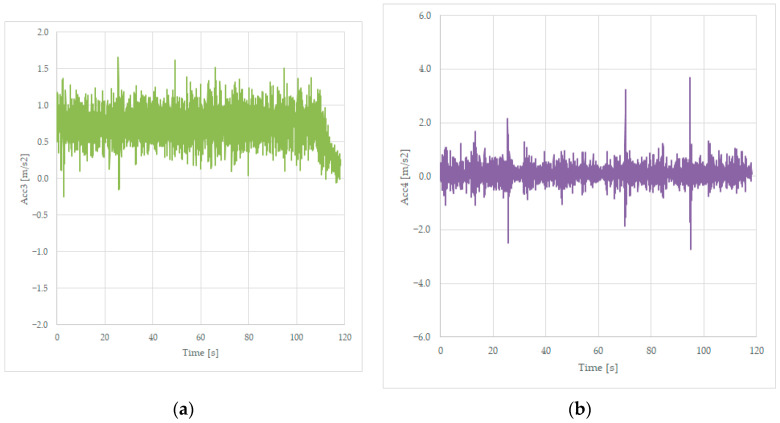
Transversal acceleration (**a**) and vertical acceleration (**b**) were recorded while running loaded, on a curve at 110 km/h, on the frame, near bogie 1.

Tests were performed, and data were recorded in the same file format as the tests from Section 3.2.1, in the Acc3 variable for the transversal acceleration of the frame of the loaded wagon near bogie 1, and in the Acc4 variable for the vertical acceleration measured on the same place on the loaded wagon, as presented in Figure 3. Graphs are presented in Figure 13.

The lateral acceleration is close to the one recorded by the sensors mounted on the axles and presented in Figure 11. But the vertical acceleration has smaller values, up to 3.6 m/s^2^, much less important than the transversal acceleration of 6 m/s^2^ for the unloaded wagon, mostly because the suspension operates better under tension. Once again, three peak acceleration values were measured, corresponding to some line irregularities that were observed in prior Figure 6b and Figure 7b.

#### 3.2.3. Acceleration Measured on the Second Bogie

**Figure 14:**
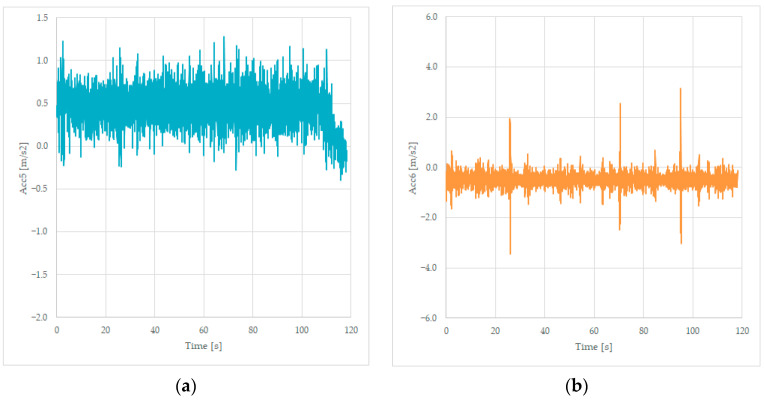
Transversal acceleration (**a**) and vertical acceleration (**b**) were recorded while running loaded, on a curve at 110 km/h, on the frame, near the bogie 2.

Similarly, we will present the last two signals measured, the transversal acceleration Acc5 and the vertical acceleration Acc6, measured on the frame of the wagon, near the bogie 2. The values are stored in columns 6 and 7, as presented in Table 1, and the graphs are shown in Figure 14.

The transversal accelerations measured near the second bogie are measuring average values of 0.6 m/s^2^, which is like the previously measured value based on Acc1, Acc2, and Acc3 recorded data. The vertical accelerations recorded as Acc6 maintain negative values but are less important than in the previous case of an unloaded wagon because of the suspension of the wagon. Peaks are recorded in the same position as in Figure 13b, when crossing the same rail joints.

#### 3.2.4. Interpretation of the Measurements

Similarly, in the analysis of the loaded wagon, the measurements must be compared and analyzed to determine the lateral stability of the wagon, the derailment speed limit prediction, and the vertical load of each bogie.

**Figure 15:**
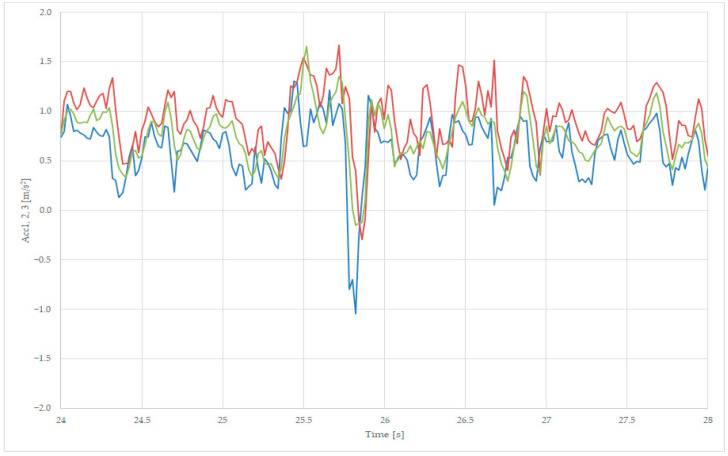
Comparing transversal acceleration, sec 19 (axle 1—blue, axle 2—red, and body—green), for loaded wagon.

For lateral stability analysis, we superpose the transversal accelerations Acc1, Acc2, and Acc3 values in one graph, near the point of the maximum amplitude of the recorded accelerations, as presented in Figure 11 and Figure 13a. We zoom in on the points 24–28 s, as presented in Figure 15, where the variables are Acc1—blue, Acc2—red, and Acc3—green.

Figure 15 presents the maximum positive point of the first axle acceleration Acc1 at moment 25.5 s, with a value of 1.61 m/s^2^, and 25.7 s with a value of 1.62 m/s^2^, the latter being followed by a maximum negative acceleration at moment 25.8 s, with a value of −1 m/s^2^. All three accelerations have similar values, and the first axle is subject to greater negative values, as the frame has a similar value to the average value measured on the two axles, due to the suspension’s tampering action.

### 3.3. General Considerations about the Acceleration’s Values

For the data recorded when running an unloaded wagon, on a curve, with a speed of 125 km/h, Table 4 contains the average value, the Real Mean Square RMS, the minimum, and the maximum values.

For the data recorded when running a loaded wagon, on a curve, with a speed of 110 km/h, Table 5 contains the average value, the RMS, the minimum, and the maximum values.

The average values must be the transversal acceleration of the vehicle in a curve, and values must be similar for all transversal accelerations Acc1, Acc2, Acc3, and Acc5.

The min–max variations depend on the suspension of the line for Acc1 and Acc2 and are highly influenced by the wagon suspension on Acc3 to Acc6.

## 4. Discussion

This paper presents a study of the transversal vibrations, on the Y axis, of a standard tank wagon, on two axles and two car bodies, Zac’s type. It analyzes and compares the vibrations induced on the axles and bogie boxes of the loaded and empty wagon at the points where the major deviations from the standard dimensions of the railway were measured. The measurements were recorded on a curve on the ring from the Romanian Railway Testing Center in Făurei at a speed of 135 km/h. The analyzed data are transposed into diagrams that reflect the presence of irregularities in the railway profile as detected in the movements of the wagon.

Regarding the position in which the accelerometers were mounted, there are two accelerometers for transversal acceleration mounted on axle boxes of the frontal bogie and two pairs of accelerometers mounted on the car body, near each bogie, for both transversal and vertical acceleration monitoring. Data are collected at 50 ms (20 Hz) using an acquisition system with six active channels. Data are stored in spreadsheet files and can be further analyzed.

The comparison of axle-related accelerations and transversal acceleration, as presented in Figure 8, shows that the body acceleration is more suitable for analyzing comfort-related issues, while the second axle acceleration is recommended for the rail irregularities evaluation, which becomes more relevant in terms of accelerations with negative values, as presented in Figure 9.

The transversal accelerations present higher values for the loaded wagon, as in Table 4 and Table 5, stating that the suspension of the wagon does not operate smoothly, but the wagon under test has a simple suspension and the results are correct.

For the vertical acceleration, as seen in Table 4 and Table 5, there is a major difference between the first and second bogie measurements, especially for the unloaded wagon.

The experiment was performed using a single wagon towed by a locomotive, and the first bogie is linked to the locomotive, while the second bogie is near the free end of the wagon, thus making the vertical accelerations measured by the first sensor collecting the line release under the weight of the locomotive (max values of −3.885/5.987 m/s^2^), while the second vertical acceleration sensor records much lesser values (−2.433 m/s^2^) because the line is loaded only by the wagon.

The line’s irregularities were sensed by the vertical acceleration sensors similarly, as can be observed from Figure 6b, Figure 7b, Figure 13b and Figure 14b, where there are 3 peak values corresponding to the irregularities from the line, respectively, three dilatation joints located on the three small bridges of the curve.

From the Figure 15 analysis on a loaded wagon, we can observe that the frame acceleration Acc3 has similar values to the ones of the axle acceleration sensors Acc1 and Acc2, thus can be used to use only data provided by the frame acceleration sensor for characterizing the loaded wagon at operational speed.

For measuring the maximum value of the lateral acceleration, in a loaded and unloaded state, the maximum value of 1.705 m/s^2^ was recorded on the first axle, but all the sensors provided similar maximal values within 1.44…1.70 m/s^2^.

For measuring the maximum value of the vertical acceleration, in loaded and unloaded states, the frame measurements on bogie 1 provide 5.987 m/s^2^.

For future work, we intend to develop an electronic unit based on acceleration sensors and place two such units on the wagon near the bogie insertion point. The rough data from the sensors, as presented in Figure 6, Figure 7, Figure 13 and Figure 14, must undergo a signal processing procedure performed by the diagnosing unit of the vehicle, where, packed with the GPS and time stamp, it can be saved for offline analysis or sent immediately to the railway authority, which can decide an intervention on the rail, a revision of the vehicle, or a reduction in the maximum allowed speed on that particular track. This additional functionality of the wagon can improve safety and increase the comfort of the passengers as an option to the regular maintenance of the tracks, which is performed on a monthly basis using dedicated test vehicles.

Future research may aim to analyze the comparison between the vibrations of the two bogies based on the measured acceleration. The comparison between the accelerations measured on the car body can be the basis for the development of a method for monitoring the state of the vehicle’s primary suspension. The acceleration sensors will be connected to an electronic microcontroller able to filter the inrush acceleration and send it to the diagnosis system of the wagon, where it can be packed with a time/localization stamp and memorized.

## 5. Conclusions

The transversal vibrations of the car body of a tank wagon, both empty and loaded, were analyzed, and the results from Table 4 and Table 5 state that there are similar values in columns 2, 3, and 4. The first conclusion is that body-mounted accelerators will measure the same data as axle accelerometers. The analysis was based on instantaneous acceleration for several measurement sequences in unloaded and loaded situations. The results showed that the acceleration is about two times higher in the car body near the second bogie insertion point than in the first one. The acceleration analysis for several measurement sequences at the same speed has made visible the influence of track defects on the bogie vibrations. The study of acceleration when rolling in a curve, on the *y*-axis, shows that the centrifugal force has the greatest influence on the recorded values. All graphs have the same characteristics.

The novelty of this study is the identification of the optimal placement of acceleration sensors for online monitoring of the operation of the wagon using the tests performed with a wagon in new condition on a smooth line by recording the accelerations from different sensors placed on the wagon’s structure.

The measurements of the tank car vibrations may lead to experimental analysis and can help engineers in the wagon construction field make better suspensions. Our data can help them solve all critical limits of the axis and the bogie of railway cars. Extending this study to other wagons can let the engineers improve the mechanics of freight and passenger cars, the rise of the speed limit, within the safety limits.

## Figures and Tables

**Table 1:** Specifications of the selected acceleration sensor.

Characteristics	Values	Units
Weight	17	grams
Nominal acceleration	+/−1000	m/s^2^
Voltage sensitivity	0.4 +/− 5%	mV/ms^−2^
Frequency range	0.1 to 250	Hz
Bias voltage	2.5	V
Power supply—constant voltage	1 to 6	V
Dimensions (excl. connector)	13 × 13 × 40	mm
Polarity: positive for acceleration in the longitudinal direction		

**Table 2:** Specifications of the selected signal amplifier type MGC plus.

Characteristics	Values	Units
Sampling rates	19.2	kS/s
Channels	16	
Accuracy class	0.03	
Input for voltage measurement	+/−10.2	V
Response time (with filters)	1	ms
Input for current measurement	+/−20	mA
Max. measurement frequency range	2400	Hz

**Table 3:** Example of the recorded file as stored on the measuring amplifier.

Time	Acc1	Acc2	Acc3	Acc4	Acc5	Acc6
[s]	[m/s^2^]	[m/s^2^]	[m/s^2^]	[m/s^2^]	[m/s^2^]	[m/s^2^]
0.00	0.87	1.14	0.89	−0.07	0.60	−0.75
0.02	0.77	1.14	0.91	−0.09	0.58	−0.66
0.04	0.64	1.22	0.93	−0.03	0.46	−0.47
0.06	0.66	1.17	0.94	0.01	0.44	−0.21
0.08	0.83	1.18	1.04	−0.04	0.57	0.10
0.10	0.85	1.31	1.04	0.07	0.61	0.19
0.12	0.83	1.17	0.99	0.18	0.68	0.24
0.14	0.81	1.24	1.03	0.14	0.75	0.04
0.16	0.78	1.11	1.01	0.21	0.78	−0.36
0.18	0.75	1.12	0.94	0.13	0.77	−0.52

**Table 4:** Evaluation of the recorded data for the unloaded wagon, in m/s^2^.

Type	Acc1	Acc2	Acc3	Acc4	Acc5	Acc6
Average	0.189	0.071	−0.002	0.364	0.105	−1.780
RMS	0.218	0.139	0.114	0.613	0.402	1.781
Min.	−0.353	−0.378	−0.374	−3.885	−1.405	−2.433
Max.	0.532	0.444	0.379	5.987	1.440	−0.920

**Table 5:** Evaluation of the recorded data for the loaded wagon, in m/s^2^.

Type	Acc1	Acc2	Acc3	Acc4	Acc5	Acc6
Average	0.635	0.884	0.723	0.105	0.459	−0.472
RMS	0.678	0.918	0.758	0.346	0.509	0.561
Min.	−1.042	−0.293	−0.243	−2.700	−0.390	−3.412
Max.	1.462	1.705	1.658	3.695	1.277	3.135

## Data Availability

Data sharing is not applicable to this article.
